# X-Linked Gene Transcription Patterns in Female and Male *In Vivo*, *In Vitro* and Cloned Porcine Individual Blastocysts

**DOI:** 10.1371/journal.pone.0051398

**Published:** 2012-12-07

**Authors:** Chi-Hun Park, Young Hee Jeong, Yeun-Ik Jeong, Se-Yeong Lee, Yeon-Woo Jeong, Taeyoung Shin, Nam-Hyung Kim, Eui-Bae Jeung, Sang-Hwan Hyun, Chang-Kyu Lee, Eunsong Lee, Woo Suk Hwang

**Affiliations:** 1 Sooam Biotech Research Foundation, Seoul, Republic of Korea; 2 Department of Animal Sciences, Chungbuk National University, Cheongju, Republic of Korea; 3 College of Veterinary Medicine, Chungbuk National University, Cheongju, Republic of Korea; 4 Department of Agricultural Biotechnology, Animal Biotechnology Major, and Research Institute for Agriculture and Life Science, Seoul National University, Seoul, Republic of Korea; 5 College of Veterinary Medicine and Institute of Veterinary Science, Kangwon National University, Chunchon, Republic of Korea; University of Connecticut, USA, United States of America

## Abstract

To determine the presence of sexual dimorphic transcription and how *in vitro* culture environments influence X-linked gene transcription patterns in preimplantation embryos, we analyzed mRNA expression levels in *in vivo*-derived, *in vitro*-fertilized (IVF), and cloned porcine blastocysts. Our results clearly show that sex-biased expression occurred between female and male *in vivo* blastocysts in X-linked genes. The expression levels of *XIST*, *G6PD*, *HPRT1*, *PGK1*, and *BEX1* were significantly higher in female than in male blastocysts, but *ZXDA* displayed higher levels in male than in female blastocysts. Although we found aberrant expression patterns for several genes in IVF and cloned blastocysts, similar sex-biased expression patterns (on average) were observed between the sexes. The transcript levels of *BEX1* and *XIST* were upregulated and *PGK1* was downregulated in both IVF and cloned blastocysts compared with *in vivo* counterparts. Moreover, a remarkable degree of expression heterogeneity was observed among individual cloned embryos (the level of heterogeneity was similar in both sexes) but only a small proportion of female IVF embryos exhibited variability, indicating that this phenomenon may be primarily caused by faulty reprogramming by the somatic cell nuclear transfer (SCNT) process rather than *in vitro* conditions. Aberrant expression patterns in cloned embryos of both sexes were not ameliorated by treatment with Scriptaid as a potent HDACi, although the blastocyst rate increased remarkably after this treatment. Taken together, these results indicate that female and male porcine blastocysts produced *in vivo* and *in vitro* transcriptional sexual dimorphisms in the selected X-linked genes and compensation of X-linked gene dosage may not occur at the blastocyst stage. Moreover, altered X-linked gene expression frequently occurred in porcine IVF and cloned embryos, indicating that X-linked gene regulation is susceptible to *in vitro* culture and the SCNT process, which may eventually lead to problems with embryonic or placental defects.

## Introduction

The onset of X chromosome inactivation (XCI) is regulated by the X-inactive specific transcript (*Xist*), which coats the inactive X chromosome in *cis* and compensates for sex-linked gene dosage differences. The initiation of X inactivation occurs in early cleavage stage embryos and it continues in the trophectoderm and the primitive endoderm. The inactive paternal X is reactivated in the inner cell mass (ICM), where it occurs randomly from either the paternal or maternal X chromosome in the epiblast [1], [2]. Recent data suggest that the mechanism of XCI involves the same sequence of events in mouse and human embryos, although a species-specific timing-window for XCI initiation and establishment exists [3]. Evidence from global transcriptome analysis indicates that a large number of X-linked genes are regulated differently between the sexes in early mammalian embryos [4,5]. This transcriptional level sexual dimorphism creates differences in developmental kinetics and epigenetics between female and male embryos during preimplantation development [6]. Furthermore, sexual dimorphisms in gene expression are found in the placenta between; thus, female and male conceptuses respond differently to diet changes in the maternal environment [7]. Consequently, these differences can lead to sex-specific embryo viability under *in vitro* conditions, such as cryopreservation and stressful culture systems (i.e., nutritional or oxidative conditions), and sex-ratio skewing of offspring because of environmental factors [8–10].

**Figure 1:**
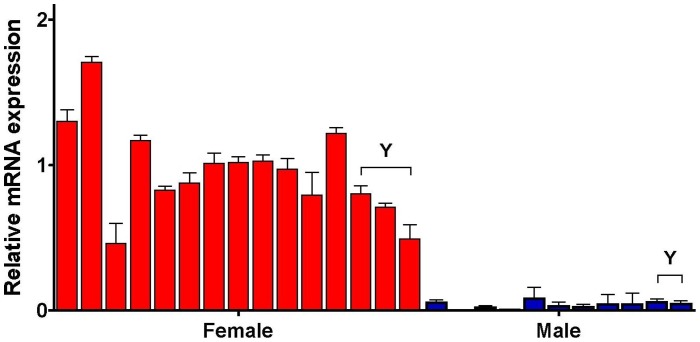
XIST mRNA expression of individual *in vivo* blastocysts. Each value derived from transcripts of the *XIST* gene in *in vivo* blastocysts derived from commercial (n = 15) and Yucatan miniature pig (n = 5), after normalization relative to *ACTB* and *18S* (internal control) genes, were compared with that of one of 26 *in vivo* blastocysts defined as 1. An underlined Y above bars indicates the blastocysts derived from Yucatan miniature pigs.

Although clones of numerous mammalian species have been successfully created using somatic cell nuclear transfer (SCNT) technology, overall cloning efficiency is still low [11]. This low efficiency appears to stem largely from incomplete reprogramming of the transferred donor cell nuclei in the oocyte cytoplasm [12]. One of the many abnormalities observed in cloned fetus and neonates is placental malformation, which results in developmental defects [13]. The regulation of X-linked and imprinted genes may be particularly susceptible to epigenetic errors during the SCNT process and *in vitro* culture [14]–[16]. Previous studies have shown that disruption of XCI in extraembryonic tissues can cause long-term growth impairment and decreased survival in clones [17],[18]. Recently, Inoue and colleagues found that cloned embryos fail to appropriately repress ectopic *Xist* expression from the active X (Xa), which in turn leads to downregulation of X-linked and autosomal genes [18]. They also showed a remarkable increase in cloning efficiency of up to 19% following the deletion of *Xist* on Xa [19]. More recently, the same research group has reconfirmed this result with RNAi-mediated knockdown of *Xist* [20]. These observations indicate that aberrant or incomplete reprogramming following SCNT leads to defects in X chromosome regulation in cloned embryos, which has a profound influence on further development.

**Figure 2:**
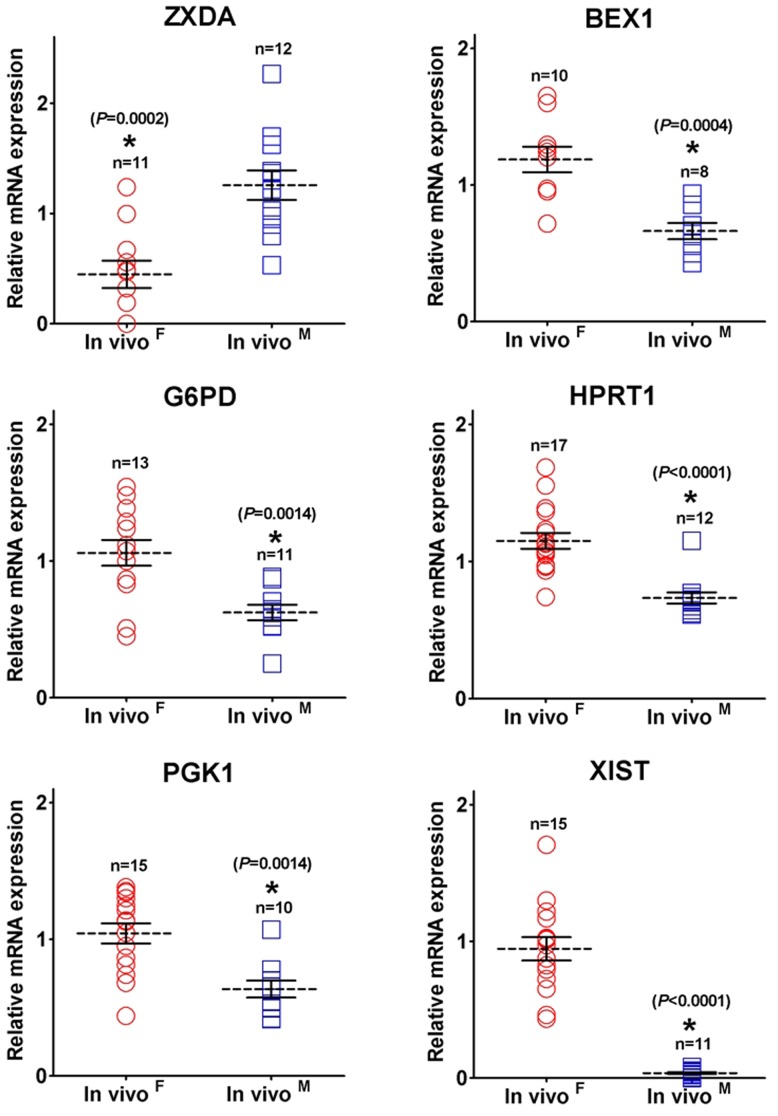
X-linked gene transcription patterns of *in vivo* porcine blastocysts. Aligned dot plots represent mRNA transcript levels for X-linked genes in female and male *in vivo* individual blastocysts. The expression values in female and male individuals are represented by red circle and blue square, respectively. A relative fold change of mRNA levels in *in vivo* male blastocysts compared with that of the *in vivo* female ones defined as 1. This data is presented as mean ± SEM. Asterisk indicates significant difference between the sexes by Student t test (*P*<0.05).

Recent studies on porcine SCNT have revealed epigenetic errors in imprinted and X-linked gene expression in obtained clones and placentas. The findings of this analysis are limited because few clones reached neonatal and adult stages [21],[22]. Less evidence at present exists regarding the regulation of X-inactivation and X-linked genes in pig embryos during preimplantation development, although these processes have been extensively studied in mice, cows, and humans. To determine the presence of sexually dimorphic transcription and the extent of the effects of *in vitro* environments on X-linked gene expression in preimplantation blastocysts, we compared X-linked gene expression between individual female and male *in vivo-*derived, *in vitro-*fertilized (IVF), and cloned blastocysts. Six X-linked genes (*BEX1*, *G6PD*, *HPRT1*, *PGK1*, *XIST*, and *ZXDA*) were selected; previous reports had identified differential expression of these genes between the sexes in early developing embryos and all were susceptible to *in vitro* environments [14],[23]. Of these genes, brain expressed X-linked protein 1 (*BEX1*), is known as candidate tumor suppressor gene and plays a role in cell cycle progression [24]. *G6PD* and *HPRT1* are related to metabolic pathways and are also involved in reactive oxygen species (ROS) detoxification [23]. Phosphoglycerate kinase (*PGK1*) is a key adenosine triphosphate-generating enzyme in glycolysis [25]. A zinc finger gene, *ZXDA* is an important regulatory complex for MHC II gene transcription [26].

**Figure 3:**
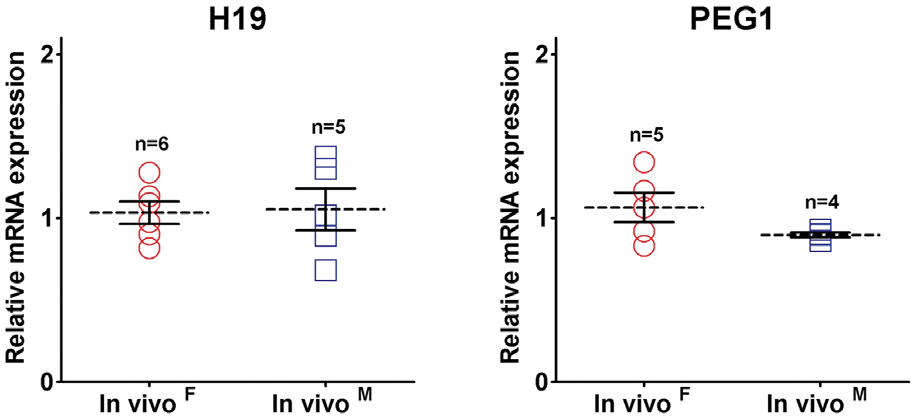
Imprinted gene transcription patterns of *in vivo* porcine blastocysts. The dot plots represent mRNA transcript levels for imprinted genes in female and male *in vivo* blastocysts. There were no differences in gene expression between the sexes of blastocysts.

In this study, we determined the presence of transcriptional dimorphisms for X-linked genes between female and male porcine embryos at the blastocyst stage. Although the average expression levels in IVF and cloned blastocysts showed the same trends in expression patterns as the *in vivo* data, impaired expression was found for some X-lined genes. To our knowledge, this is the first report of sexually dimorphic transcription in X-linked genes and the first description of how *in vitro* culture or the SCNT process influences X-linked gene regulation during preimplantation development.

**Figure 4:**
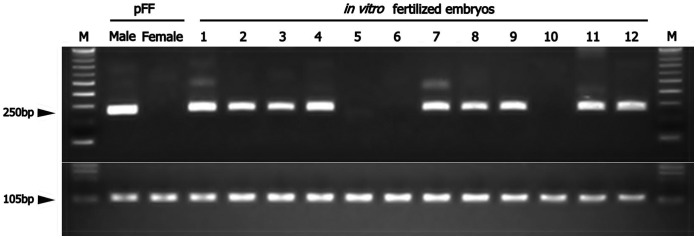
IVF embryo sexing by PCR. a representative PCR result for embryo sexing is shown. M is a 100-bp ladder as a DNA size marker. Male and female indicate M^1^ and F^1^ fetal fibroblast cell lines, respectively. 1–12 lanes are shown for IVF embryos. A single PCR product (250-bp and 105-bp) was detected for the *SRY* and the *ACTB*, respectively.

## Results

### Comparison of X-linked Gene Transcription Patterns between Female and Male *in vivo* Blastocyst

As shown in Figure 1, the genders of individual *in vivo*-derived blastocysts, which were derived from commercial (n = 21) and miniature (n = 5) breeds, were determined quantitatively by differential *XIST* expression values, as previous described [27]. This method can be used to determine the sex of an embryo, although it is not as accurate as genomic polymerase chain reaction (PCR) with sex-specific DNA sequences. Our results show that sexual dimorphisms in X-linked gene expression occurred in porcine embryos at the blastocyst stage (Figure 2). The expression levels of *BEX1, G6PD*, *HPRT1*, *PGK1*, and *XIST* were significantly higher in female than in male blastocysts (*P*<0.01), while *ZXDA* levels were significantly higher in males than in females (*P* = 0.0002). *XIST* transcripts were approximately 25-fold higher in female compared with male embryos, while the other genes exhibited approximately 1.5-fold differences between the sexes. In addition, no difference in *XIST* mRNA levels was observed between miniature and commercial breeds (female, *P* = 0.067 and male, *P* = 0.347). *ZXDA* transcripts exhibited relatively higher heterogeneity among individuals than the other genes tested. The present experiments were also conducted to determine if autosomal genes showed similar differences between the sexes in their relative transcription levels. However, two autosomal genes (two imprinted genes: *H19* and *PEG1*; Figure 3) and three housekeeping genes (*RN18S*, *ACTB*, and *GAPDH*; data not shown) showed no differences between the sexes. Thus, sex-biased transcription patterns were not the result of variation in cDNA samples. In addition, transcript levels were quite variable among individual embryos in X-linked genes compared with imprinted genes, but these differences were not statistically significant. Taken together, these results indicate that porcine embryos showed transcriptional sexual dimorphisms in X-linked genes at the blastocyst stage.

**Figure 5:**
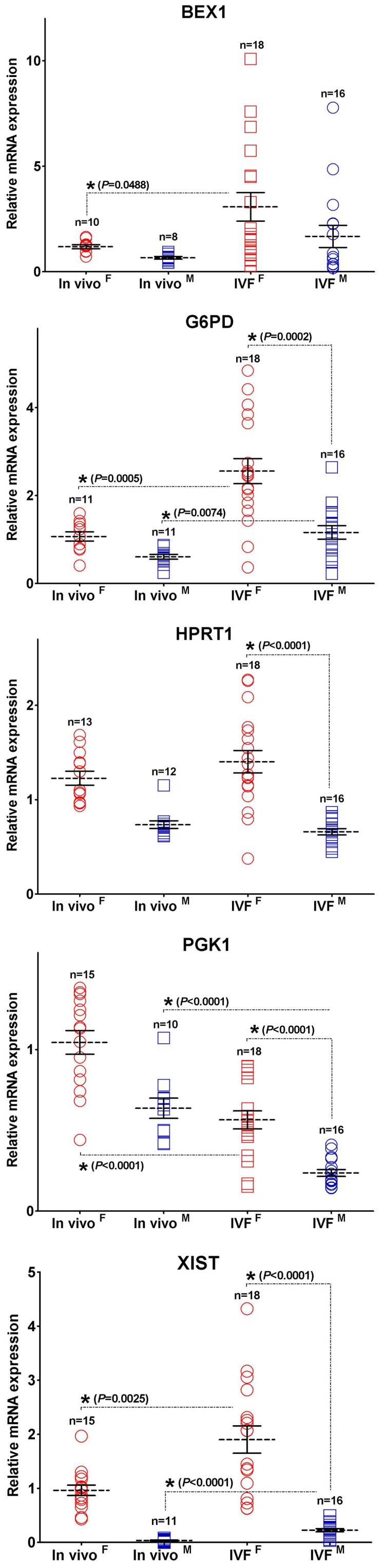
X-linked gene transcription patterns of IVF blastocysts. The dot plots of mRNA transcript levels for X-linked in female and male *in vivo* and *in vitro* fertilized (IVF) blastocysts. Asterisks indicate significant difference between *in vivo* and IVF embryos of each sex as well as between female and male IVF embryos. A relative fold change of mRNA levels of female and male IVF blastocysts compared with that of the *in vivo* female ones defined as 1. Asterisks indicate significant difference between *in vivo* and IVF blastocysts of each sex as well as between female and male IVF embryos (*P*<0.05).

**Figure 6:**
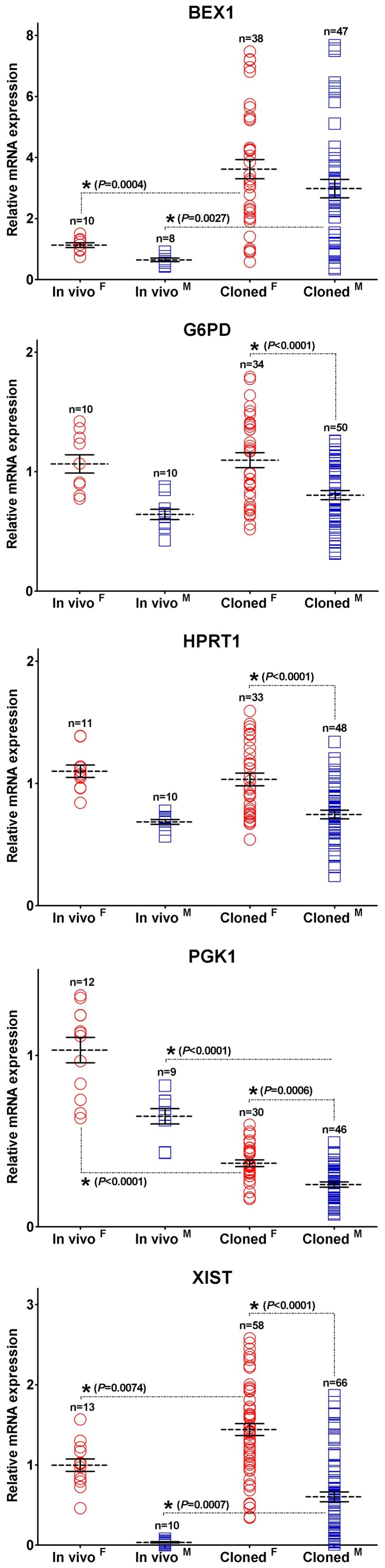
X-linked gene transcription patterns of cloned blastocysts. The dot plots of mRNA transcript levels for X-linked in female and male *in vivo* and cloned blastocysts. Other details are as described in the legends to **Figure 5**.

### Comparison of X-linked Gene Transcription Patterns between Female and Male IVF Blastocysts

To determine whether sex-related transcriptional differences also occur in blastocysts produced in an *in vitro* culture environment, the expression levels of six X-linked genes were also assessed in IVF embryos. For comparison, sexing was performed on a total of 32 IVF blastocysts (female n = 18, male n = 16) using PCR assays (Figure 4). Figure 5 shows that significant differences were observed in average mRNA levels for *G6PD*, *HPRT1*, *PGK1*, and *XIST* between female and male IVF blastocysts. Although the relative abundance of *BEX1* transcripts showed a similar tendency, no statistically significant patterns were observed. These X-linked gene transcription patterns were more variable in female compared to male IVF blastocysts. For most of the assessed genes, the difference in the average expression level was approximately 2-fold between the sexes as well as between *in vitro-* and *in vivo*-derived blastocysts. In particular, *XIST* mRNA expression was approximately 5-fold higher in female than in male IVF blastocysts and was higher in male IVF than in male *in vivo*-derived blastocysts. For *G6PD* and *XIST* transcripts, increased expression was observed in all of the female and male IVF blastocysts compared with *in vivo* blastocysts. *PGK1* genes were transcribed at lower levels in IVF than in *in vivo* blastocysts. For *XIST*, the range of the fold-change (15- to 60-fold) between the sexes allowed us to readily distinguish the sex of *in vivo*-derived blastocysts. However, IVF blastocysts did not demonstrate a high enough fold-difference (range, 1.2- to 8-fold) for sex determination on the basis of *XIST* transcript levels. This difference can be attributable to increased expression in IVF embryos compared with their *in vivo* counterparts, especially in males. Additionally, expression data for *ZXDA* mRNA were only available from a few examples, perhaps because the amount of starting material in each individual was low. These data were therefore excluded from further experimental and statistical analyses. These results suggest that the expression of X-linked genes in IVF blastocysts may follow the same trend as observed in their *in vivo*-derived counterparts.

**Figure 7:**
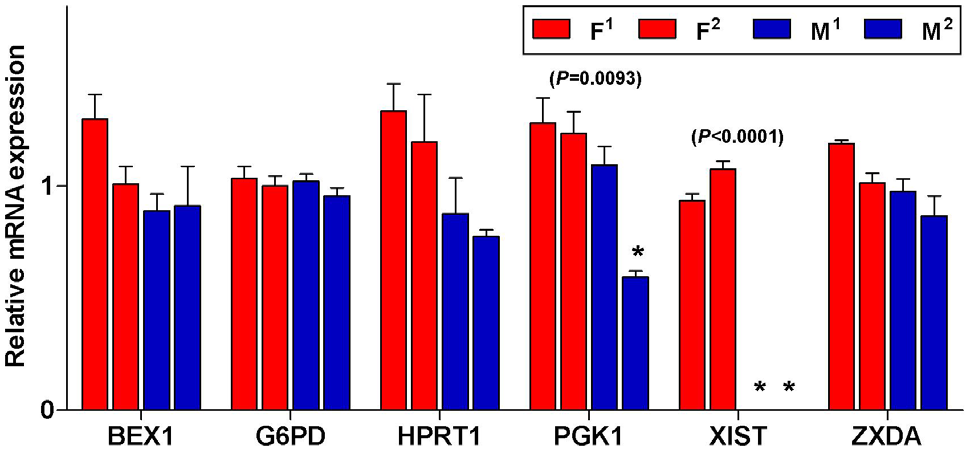
X-linked gene transcription patterns of pFF cell lines. Asterisks indicate significant difference between the different cell lines (*P*<0.01).

### Comparison of X-linked Gene Transcription Patterns between Female and Male Cloned Blastocysts

To determine if SCNT affected X-linked gene regulation, levels of X-linked gene transcripts were analyzed in blastocysts cloned from female and male adult ear fibroblasts (AF). Figure 6 shows that the average levels of *G6PD*, *HPRT1*, *PGK1,* and *XIST* transcripts were significantly higher in female than male cloned blastocysts. *BEX1* did not differ significantly between the sexes. *BEX1* and *XIST* transcript levels showed larger inter-embryo fluctuations in the cloned embryos than in IVF embryos. The levels of *G6PD* and *HPRT1* transcripts were relatively stable in both sexes of cloned blastocysts and were comparable with *in vivo* levels. Abnormal expression patterns for several genes were observed in most of the cloned blastocysts; *BEX1* and *XIST* were upregulated and *PGK1* was downregulated. This contrasted with the observations of the IVF blastocysts, in which variable gene expression was only exhibited by a small subset of individuals. The expression levels of other genes were within the normal range. These results indicate that transcriptional differences in X-linked genes existed between female and male cloned blastocysts, even though individual cloned embryos of both sexes displayed abnormal expression levels and inter-embryo variability was observed in the expression of several genes.

**Table 1:** In Vitro Development of the Male and Female Cloned Embryos.

Group		No. reconstructed		No. Cleaved (%)†		No. blastocyst (% of cleaved)	
IVF		562	(n = 8)	328	(62.4±4.6)	161	(32.7±4.7)^ a^
NT	F^1^	328	(n = 7)	188	(58.5±4.9)	53	(16.1±1.8)^ c^
	F^2^	329	(n = 8)	194	(61.2±2.6)	46	(14.5±1.8)^ d^
	M^1^	294	(n = 6)	164	(55.6±4.2)	34	(11.3±2.2)^ d^
	M^2^	260	(n = 5)	137	(53.9±3.8)	48	(18.1±1.6)^ b^
NT ^Sc^ ‡	F^1^	312	(n = 7)	172	(55.4±2.5)	71	(22.9±1.7)^ b^
	F^2^	310	(n = 8)	183	(59.1±3.5)	90	(30.7±3.7)^ a^
	M^1^	296	(n = 6)	171	(57.8±5.1)	63	(21.4±5.4)^ b^
	M^2^	328	(n = 6)	191	(58.8±2.7)	85	(26.1±2.5)^ a^

† the cleavage rate was counted after 2 days in culture.

‡ Sc; scriptaid, which were treated for 14 hr after post-activation.

a−d Values with different superscripts within each column are significantly different, *P*<0.05.

### Changes in X-linked Gene Transcription Patterns in Cloned Embryos by Treatment of Scriptaid, a Histone Deacetylase Inhibitor, after SCNT

We explored if treatment with Scriptaid (Sc), a histone deacetylase inhibitor (HDACi), could improve reprogramming efficiency following SCNT and thus ameliorate aberrant X-linked gene transcription patterns in cloned embryos. To compare differences in expression between cell lines for X-linked genes, two fetal fibroblast (FF) cell lines for each sex were used as donor nuclei for SCNT. As expected, among the cell lines, equivalent expression was observed in most of the X-linked genes tested, with the exception of *PGK1*, which showed a significant decrease in the M^2^ line compared with the other lines (Figure 7). *XIST* was exclusively expressed in females and was over 10,000 times higher than in males. Thus, these cell lines appear to have achieved compensation of X-linked gene dosage between females and males. Although no differences were observed in the cleavage rates of cloned embryos produced by any of these cell lines, the blastocyst rate differed significantly among them (from 11.3% to 18.1%, *P*<0.05). Groups treated with Sc showed markedly increased blastocyst rates compared with untreated groups, although the range of the influence of this treatment on the cloned embryos differed among cell lines (*P*<0.05, Table 1). Therefore, the results confirmed that the Sc treatment enhanced the developmental potential of cloned porcine embryos, as previously described [28]. However, after SCNT, the different cell lines had different *in vitro* developmental potentials and the higher blastocyst rate in the donor cell line was not the same for full-term embryos, indicating that *in vitro* developmental potential in the different cell lines did not correlate with cloning efficiency (Table 2).

**Table 2:** In Vivo Development of the Cloned Embryos.

Cell line	No. experiments	No. embryos transferred	No.(%) pregnancy*	No. live offspring†
F^1^	6	635	3 (50)	18
M^1^	6	719	3 (50)	11
M^2^	5	596	2§ (40)	–

* an initial pregnancy diagnosis was examined via ultrasonography at 27 to 30 days after embryo transfer.

§ the fetuses were aborted from two recipients at Day 55 to 60.

† all of the cloned piglets were vaginally delivered.

**Figure 8:**
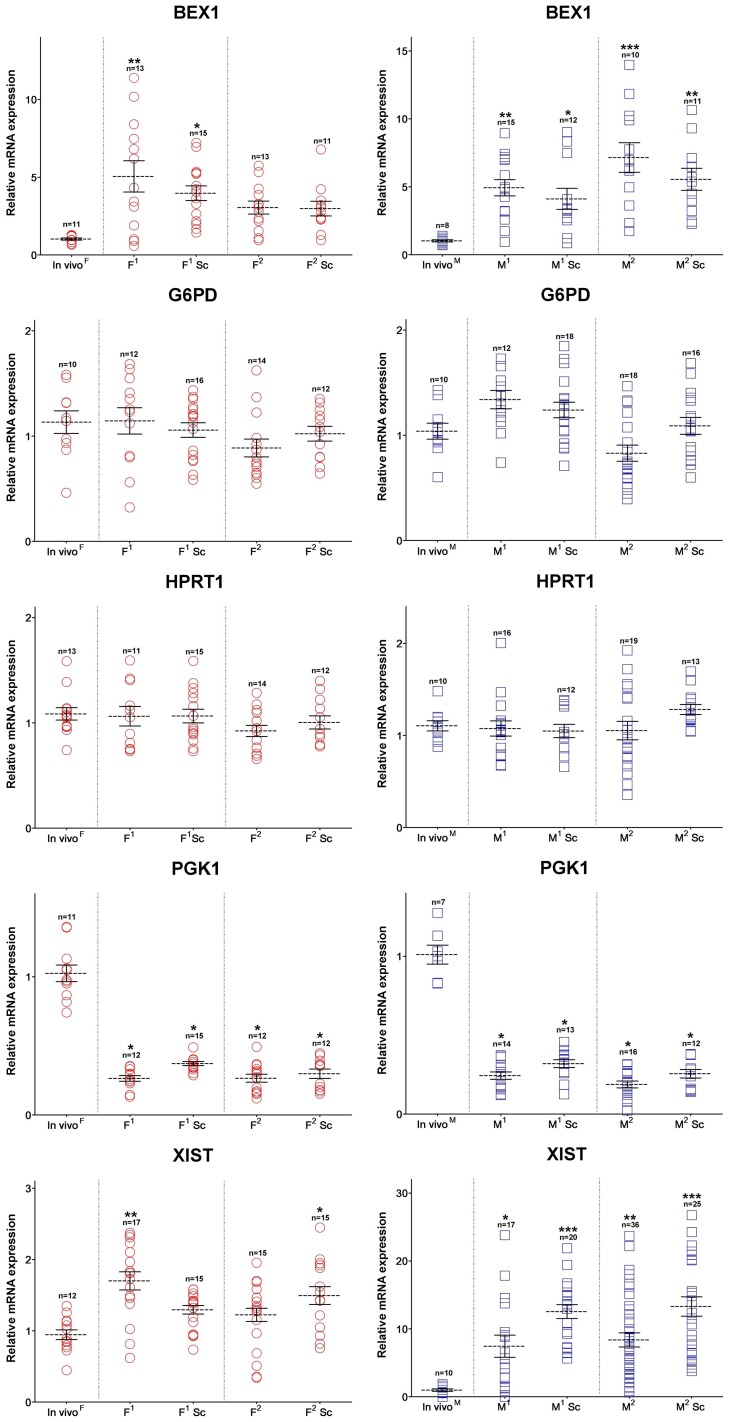
X-linked gene transcription patterns of female and male porcine cloned blastocysts by treatment of Scriptaid, a HDACi after SCNT. A relative fold change of mRNA levels of female (Left panel) and male (right panel) cloned blastocysts compared with that of the *in vivo* female ones defined as 1. Asterisks indicate significant difference between *in vivo* and cloned groups (* *P*<0.05; ** *P*<0.01; *** *P*<0.001).

Figure 8 shows that cloned embryos from lines of the same sex had significantly different average expression levels for two genes, *XIST* and *BEX1*. This may have been caused by heterogeneity at different ranges. Note that the groups exhibiting high variability tended to have reduced *BEX1* transcripts after treatment with Sc. In contrast, *XIST* transcript levels increased in the Sc-treated groups compared with non-treated groups, except for the F^1^ group. For *G6PD*, *HPRT1,* and *PGK1*, the mRNA expression levels did not differ between the Sc-treated and non-treated groups. The Sc treatment clearly seems to have increased the developmental potential of cloned porcine embryos. However, a similar effect on X-linked gene expression was only obtained for a few genes, and genes that had increased or decreased transcript levels in cloned blastocysts showed no changes in response to Sc.

## Discussion

The present study showed that female and male porcine blastocysts that were produced *in vivo* and *in vitro* displayed sex-biased transcription patterns in the selected X-linked genes. Moreover, aberrant X-linked gene expression occurred frequently in embryos that were produced *in vitro* before implantation, although the same general trend in expression patterns was seen in both types of embryos. Recent studies on transcriptional profiling have suggested that most X-linked genes display not only sex-related transcriptional differences but are also involved in the regulation of autosomal gene expression in preimplantation embryos [5]. Clear evidence exists that impaired *Xist* regulation occurs in cloned embryos and confers an increased risk for placental defects and neonatal death in mammalian cloned embryos [18],[29]. Two studies by the same research group have supported the idea that the suppression of *Xist* upregulation, by knockout or RNAi knockdown techniques, has apparent global effects not only on the X-chromosome but also on autosomal expression in cloned mouse embryos [19],[20].

**Figure 9:**
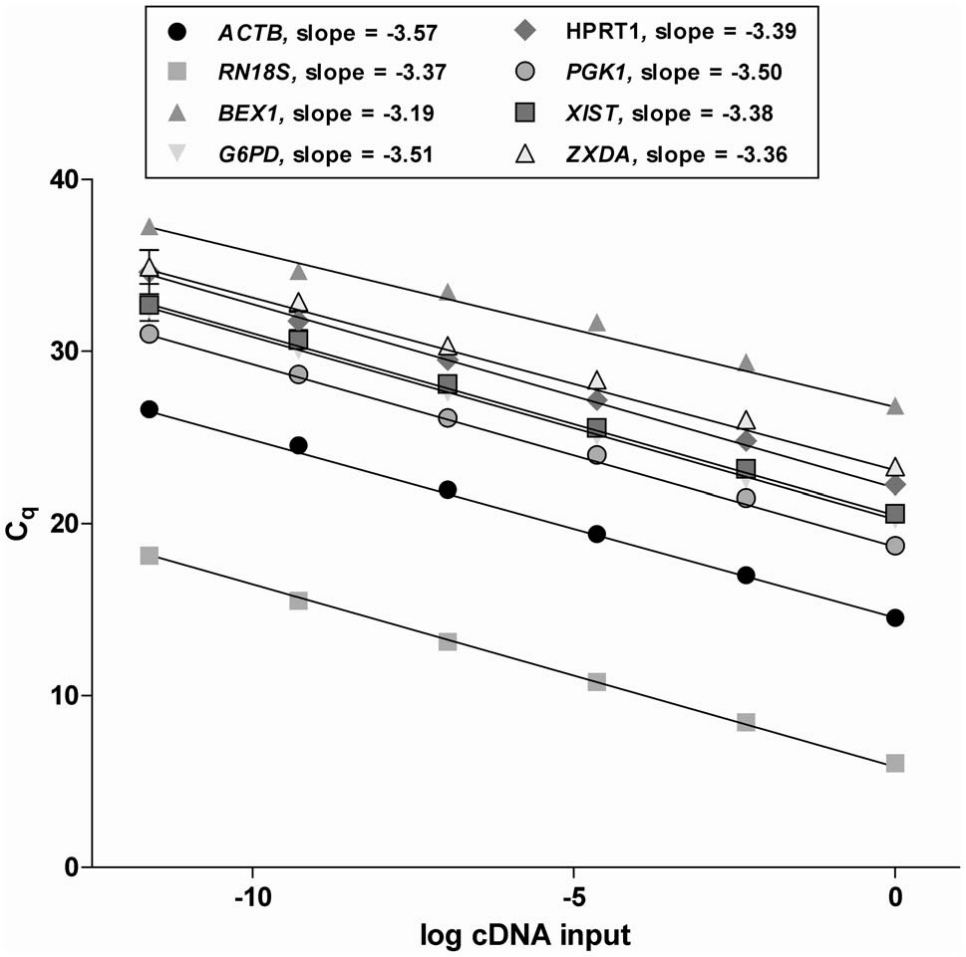
Calculation of **amplification efficiencies.** qPCR efficiencies of reference (*ACTB* and *RN18S*) and target genes (*BEX1*, *G6GD*, *HPRT1*, *PGK1*, *XIST* and *ZXDA*). The C_q_ was plotted against the log of the initial quantity of template for each dilution of cDNA (50 ng–16 pg, n = 3).

**Table 3:** Primer sequences for qRT-PCR.

Gene	Primer sequence 5′-3′	Gene Access no.	Length (bp)
*BEX1*	F: AGCCACAGGCAAGGATGAGA	XM_003135280.1	90
	R:TCAACTGCTTTTCCCTCAGCTT		
*G6PD*	F:TTCTTTGCCCGCAACTCCTA	XM_003135508.1	90
	R:GCGTTCATGTGGCTGTTGAG		
*HPRT1*	F:CATTATGCCGAGGATTTGGAA	NM_001032376.2	90
	R:CTCTTTCATCACATCTCGAGCAA		
*PGK1*	F:GCTCGGGCTAAGCAGATTGT	AY677198.1	84
	R:CCATGAGGGCTTTGGTTCCT		
*XIST*	F:TGTGGGCTCTTGTGTGTTTGTAA	EF619477.1	92
	R:TCTGCAATGCTTATTTTGGTAGCT		
*ZXDA*	F:GGCTGTTGTGCCAGGTTCTC	XM_003135136.1	90
	R:GCATCGGCTTTTCCAAGTGT		
*RN18S*	F:ACAAATCGCTCCACCAACTAAGA	NR_002170.3	90
	R:CGGACACGGACAGGATTGAC		
*ACTB*	F:GTGGACATCAGGAAGGACCTCTA	U07786	137
	R:ATGATCTTGATCTTCATGGTGCT		

Our data indicate that X-linked gene expression is significantly higher in female than in male *in vivo* and *in vitro* porcine blastocysts, which is consistent with previous findings [14],[23],[30],[31]. Evidence has shown that molecular sexual dimorphism in early stage embryos, before gonadal differentiation, leads to differences in developmental kinetics. Although conflicting results exist, male embryos are generally accepted to grow faster than female embryos during preimplantation development [9]. Under our experimental conditions, no apparent differences were found in speed or developmental competence between the sexes of *in vitro-*produced IVF and cloned embryos, but a skewed sex ratio toward females was observed in *in vivo* embryos that recovered from Yucatan recipients. Kaminski and colleagues suggested that pig embryonic growth is influenced by the uterine environment and not by fetal sex during preimplantation development [32]. A recent study demonstrated that female and male mouse conceptuses respond differently to the maternal environment and that the murine placenta reveals sex-biased transcription [7]. Therefore, such a sex-related phenotypic consequence that is present in early embryos could vary in its response to different environmental conditions.

Numerous studies have reported that suboptimal *in vitro* culture conditions or the SCNT process affect changes in X-linked gene expression and methylation in preimplantation embryos, which in turn can lead to long-term effects [15],[19],[33]. Our results show that both IVF and cloned embryos exhibit aberrant expression, with either up- or downregulation, for several genes, although the average levels of all X-linked gene mRNAs that were tested showed sex-specific expression. Also, somewhat distinctive patterns of gene expression were observed between IVF and cloned embryos, as well as between the sexes. Among these genes, *BEX1* exhibited variable gene expression in only a small subset of IVF embryos, while others fell within the normal range. This was in contrast to observations in cloned blastocysts in which variable gene expression was observed in a large subset of individuals, suggesting that dysregulated *BEX1* may be largely due to incomplete reprogramming following the SCNT process, rather than *in vitro* culture. Previous studies on haploid mouse parthenotes have suggested that upregulated *Bex1* expression may affect commitment to the trophectoderm lineage, which in turn could arrest development [34],[35]. Thus, determining if the observed aberrant expression of *BEX1* can influence further embryonic development in cloned porcine embryos will be interesting. The data showed relatively stable expression patterns for *G6PD* and *HPRT1* in IVF and cloned blastocysts. Levels of *G6PD* and *HPRT* transcripts were also higher in female blastocysts than in males, but only *HPRT* mRNA levels in IVF and cloned embryos were comparable to those in *in vivo* embryos. *G6PD* showed upregulated expression in IVF embryos of both sexes compared with their *in vivo* counterparts. This observation is consistent with a previous report showing increased expression of this gene in bovine IVF embryos [14]. Repressed *Pgk1* expression in cloned mouse embryos was found by Fukuda et al. [36], which is consistent with our results. However, we also found that *PGK1* was consistently downregulated in IVF embryos, indicating that aberrant expression of this gene may not be solely due to the SCNT process but may be attributable to *in vitro* cultures. Such downregulation of the glycolysis-related *PGK1* gene in both types of *in vitro* embryos may be due to our culture conditions, when glucose in the PZM3 media was depleted.

The reported onset of compensation for the *Hprt* and *Pgk1* gene dosages between XX and XY mouse embryos before the blastocyst stage [25] is in contrast to our result of an apparent sex-biased difference in porcine blastocysts for the expression of most of the tested X-linked genes. These results indicate that differences in timing to acquire compensation for X dosages may exist across mammalian species. Upregulated *XIST* expression was observed in both IVF and cloned embryos. Higher *Xist* expression in cloned embryos provides evidence that ectopic *Xist* expression from the Xa leads to abnormal XCI and is responsible for genome-wide downregulation [19]. Despite upregulated *XIST* expression in IVF and cloned embryos, we could not find repressed patterns for the other X-linked genes, except for *PGK1*, which may respond to environmental factors like the *in vitro* culture or manipulations. In the mouse, *Xist* is initially imprinted from the paternal allele at the 4–8-cell stage, but is detectable in bovine, porcine, and human preimplantation embryos from the 8- to 16-cell stage onward [27],[37],[38]. Thus, in other mammals, including pigs, the onset of XCI appears to begin later than in mice. Taken together, these data suggest that in female porcine embryos in which the *XIST* gene may not be functional, XCI, as a dosage-compensation mechanism, does not occur until the blastocyst stage.

Gene expression profiles have been reported to differ both among and within individual clones with different types of donor cells [39]. Also, imprinted gene expression has been shown to be variable in the placentas of deceased and surviving clone piglets [22]. Therefore, a large degree of the variability in expression, particularly in *BEX1* and *XIST*, among individual cloned blastocysts may be attributable to abnormal epigenetic reprogramming that occurs in a random manner. Such incomplete reprogramming reflects the extremely low efficiency of SCNT. Although overall, the IVF embryos had normal ranges of expression, noticeable gene discordance occurred in a small proportion of individuals, especially in females. These differences can be interpreted as being the effects of chromosomal abnormalities that resulted from polyspermy, which usually occurs in the porcine IVF system [40]. This assumption is supported by the observation that increased expression of X-linked genes can occur as a response to both changes in ploidy and to the number of active X chromosomes (Xa) [41]. Nevertheless, we cannot rule out the possibility that female embryos may be more sensitive to environmental conditions than male embryos.

Treatment of cloned embryos with histone deacetylase inhibitors (HDACi) was recently shown to improve the success rate of development to term in several species [28] [42]. However, this effect may not occur in X-linked gene transcription patterns in cloned embryos with TSA treatment [19]. We found that the blastocyst rate was consistently increased in all Sc treatment groups but relatively little improvement was observed in X-linked gene expression rates, with some exceptions. For *BEX1* transcription, reduced variation occurred in treated groups compared to control groups, but higher levels were still found in cloned embryos compared with their *in vivo* counterparts. Similar results were observed for the *XIST* gene in only one treated group, whereas variation in *XIST* gene expression was notably higher in the other treated groups than in the control groups. These results indicate that not all X-linked gene expression in cloned embryos responded to Sc treatment in the same manner; moreover, the effectiveness of this treatment during the SCNT process may be limited to a few genes. Similar findings were reported by Whitworth et al. [43], with increased *XIST* expression in porcine IVF embryos and up- or downregulated expression in male cloned embryos from *ex vivo* collections compared with *in vivo* controls. Unfortunately, because this study was performed using pooled embryo cDNA samples of mixed sex, making a direct comparison among embryos for this gene is difficult. Nevertheless, this finding appears to indicate that aberrant regulation of X-linked gene expressions can occur within a short exposure time to *in vitro* environments, even before the fertilization process, or throughout the SCNT process. Additionally, note that porcine IVM systems can lead to epigenetic defects in oocytes that mature *in vitro,* which may vary greatly among individuals [44]. Considering the possibility of misinterpretation due to individual variation in X-linked gene expression in *in vitro*-produced IVF and cloned embryos, transcriptional analyses of individual embryos may be necessary to gain a more robust measure of gene expression in preimplantation embryos. This will not only advance our understanding of the reprogramming process, but will also permit more accurate predictions of the factors affecting the *in vitro* environments of developing embryos. Moreover, a more refined analysis, such as RNA FISH for *XIST*, is needed to determine XCI patterns during embryonic development and to clarify how XCI occurs in cloned embryos.

## Materials and Methods

### Ethics Statement

The pig experiments were carried out in strict accordance with the recommendations in the Guide for the Care and Use of Laboratory Animals of the National Veterinary and Quarantine Service and were supervised by Gyeonggido Livestock and Veterinary Service. Each study was approved by the animal ethics committee of Sooam biotech research foundation (license number AEC-20081021-0001). Porcine ovaries were provided by the regional slaughterhouse (Hyup-Shin, Anyang, Korea).

### *In vitro* Maturation (IVM)

Ovaries were collected from prepubescent gilts at a local slaughterhouse and transported to the laboratory in 0.9% (w/v) NaCl supplemented with 100 mg/ml streptomycin sulfate (Amresco, Solon, OH) within 1 h at 37°C. Cumulus–oocyte complexes (COCs) were obtained from follicles that were 3–6 mm in diameter using 18-gauge microneedles. Oocytes possessing an evenly granulated cytoplasm and a compact surrounding cumulus mass were collected and washed twice with TL–HEPES–PVA medium (Tyrode’s lactate–HEPES medium supplemented with 0.01% polyvinyl alcohol). After washing, 40–50 COCs were transferred to 500 µl of IVM medium (TCM-199; Invitrogen, Carlsbad, CA) supplemented with 10 ng/ml epidermal growth factor (EGF), 1 µg/ml insulin (Sigma-Aldrich, St. Louis, MO), 4 IU/ml eCG (Intervet, Boxmeer, The Netherlands), hCG (Intervet), and 10% (v/v) porcine follicular fluid (pFF). After 22 h of culture, the COCs were transferred to an IVM medium without hormones and cultured for an additional 22 h at 38.5°C in an atmosphere containing 5% CO_2_ and 100% humidity.

### *In vitro* Fertilization (IVF)

Fertilization was performed as described in our previous study [27]. At 42 h of IVM, 15–20 denuded MII oocytes were placed in 40 µl drops of modified Tris-buffered medium (mTBM) that had been covered with warm mineral oil in a 60-mm dish. Fresh semen ejaculated from a Duroc boar was supplied by DARBY A.I. center (Chungju, South Korea). The semen sample was washed twice by centrifugation at 350×*g* for 3 min in phosphate-buffered saline (PBS). The sperm pellet was then resuspended and adjusted to a concentration of 1×10^5^ sperm/ml. The appropriate concentration of sperm was introduced into the oocyte-containing medium drop and the cells were incubated for 6 h at 38.5°C. After fertilization, excess spermatozoa were removed from oocytes by a repetitive pipetting action, and fertilized oocytes were washed three times in a culture medium (PZM3) containing a 1% nonessential amino acid/minimum essential medium solution.

### Nuclear Transfer

Briefly, adult fibroblast cells were obtained from abdominal skin biopsy and fetal fibroblast (pFF) cells were obtained from a day 27 pregnant Yucatan minipig that had mated naturally. The pFF cell lines, except for F^2^, were primarily characterized by the success rate of full-term development following SCNT (Table 2). The adult tissue samples were cut into small pieces (approx. 1 mm) with a scalpel. Then, the dissected tissues were cultured in Dulbecco’s modified Eagle’s medium (DMEM; Gibco-BRL, Grand Island, NY) with 10% fetal bovine serum until confluent; cells were frozen in DMEM with 10% fetal calf serum (FCS) and 10% dimethyl sulfoxide. Prior to use as nuclear donor cells, cells were thawed and cultured for 2–5 days in DMEM with 10% FCS. Nuclear transfer was performed as was previously described by Song et al. [45]. Enucleation was carried out in TL–HEPES supplemented with 0.4% bovine serum albumin (BSA) and 5 mg/ml cytochalasin B. Denuded oocytes were enucleated by aspirating the polar body and MII chromosomes by an enucleation pipette (Humagen, Charlottesville, VA). After enucleation, a donor cell was introduced into the perivitelline space of an enucleated oocyte. Fusion of injected oocytes was induced in fusion medium (280 mM mannitol, 0.001 mM CaCl_2_, and 0.05 mM MgCl_2_) by two DC pulses (1-s interval) of 2.0 kV/cm for 30 µs using a BTX-Cell Manipulator 200 (BTX, San Diego, CA). After fusion, oocytes were incubated for 1 h in TL–HEPES. The reconstructed oocytes were activated by an electric pulse (1.0 kV/cm for 60 µs) in activation medium (280 mM mannitol, 0.01 mM CaCl_2_, 0.05 mM MgCl_2_), followed by 4 h of incubation in PZM3 medium containing 2 mmol/l 6-dimethylaminopurine. Embryo transfers were performed at a research farm (Department of Livestock Research, Gyeonggi Veterinary Service, Korea). Approximately 100 reconstructed oocytes were surgically transferred into the oviducts of naturally cycling sows (approx. 9 months old) on the first day of standing estrus. Pregnancies were confirmed by ultrasound on day 30 (day 0 was the day of SCNT). All of the cloned piglets were delivered naturally. Despite only relatively mild abnormalities, curled toes on the front feet were occasionally observed in male cloned piglets but they usually disappeared before the weaning period.

### Scriptaid Treatment

Reconstructed oocytes were treated with 500 nM Sc for 14 h after post-fusion activation, according to a previous study [28].

### *In vitro* Culture (IVC)

About 50–80 fertilized or post-activated oocytes were cultured in 4-well dishes containing 500 µl of PZM3 for 168 h. Embryo culture conditions were maintained at 38.5°C in an atmosphere containing 5% CO_2_, 5% O_2_, and 100% humidity.

### Recovery of *in vivo* Blastocysts

Crossbred (LargeWhite×Landrace) and Yucatan miniature gilts observing first standing estrus (day 0) were mated with a mature Duroc boar and Yucatan miniature boar, respectively. Seven days later, they were slaughtered at a local abattoir and their reproductive tracts were excised. Blastocysts were collected after the uteri were flushed twice with 50 ml of PBS containing 1% BSA. Within 30 min, mRNA was directly isolated from recovered blastocysts and used to synthesize cDNA.

### mRNA Synthesis and Quantitative PCR

Embryos that reached the blastocyst stage on day 7 with good morphological features were selected and treated with Tyrode's acid to remove the zona pellucida prior to use for mRNA isolation. mRNA was immediately extracted using the Dynabeads mRNA Direct Kit (Dynal Biotech ASA, Oslo, Norway) according to the manufacturers’ instructions. cDNA synthesis was performed with the High Capacity cDNA Reverse Transcription Kit (Applied Biosystems, Foster City, CA) according to the manufacturers’ instructions. Using a final volume of 20 µl, synthesis was carried out at 37.5°C for 60 min and samples were subsequently incubated at 95°C for 5 min to inactivate reverse transcriptase. To minimize the effect of variability in individual sample quality, the amplification yield for each sample was analyzed using quantitative real-time PCR analysis with two housekeeping genes, *ACTB* and *RN18S*. Prior to use in the experiment, cDNA samples with similar threshold cycle values were frozen. All gene tested Amplification and detection were carried out with the ABI 7300 Real-Time PCR System (Applied Biosystems) using a Power SYBR Green PCR master mix (Applied Biosystems) under the following conditions: 95°C for 15 min, 40 cycles of denaturation at 95°C for 15 s, and annealing at 60°C for 60 s. All of the threshold cycle (C_T_) values of the tested genes were normalized to *ACTB* and *RN18S* expression, and relative expression ratios were calculated using the 2^–ΔΔC^_T_ method. The present data were expressed as average of 2^−ΔΔC^_T_ values which were normalized with the C_T_ for *ACTB* and *RN18S*. Specificities of all of the designed primers used in this study were confirmed via sequencing analysis (Table 3). Three to five independent experiments were performed with each replicate containing 5–7 individual blastocysts. Amplification efficiency (E) was calculated using the linear regression slope of a 5-fold dilution series with 6 steps (50 ng–16 pg) by the following equation: E = 10^(−1/slope)^, the average C_T_ values obtained from each dilution were then plotted against the logarithm of input amount of starting cDNA. These slope values showed in the range of −3.57 to −3.19 and high amplification efficiencies of 1.93 for *ACTB*, 1.98 for *RN18S*, 2.05 for *BEX1*, 1.92 for *G6PD*, 1.97 for *HPRT1*, 1.93 for *PGK1*, 1.97 for *XIST* and 1.98 for *ZXDA* (Figure 9).

### Embryo Sexing

Genomic DNA from individual blastocysts was extracted from resultant lysates, in which Dynabeads–mRNA complexes were pre-cleared using the Dynabeads DNA DIRECT Kit (Invitrogen, Carlsbad, CA) according to the manufacturer's instructions. The following primer sets were used: 5′-CTGGGATGCAAGTGGAAAAT-3′ (forward) and 5′-GGCTTTCTGTTCCTGAGCAC-3′ (reverse) for *SRY*; 5′-TGGACATCAGGAAGGACCTC-3′ (forward) and 5′-GTGCCAGGGCTGTGATCT-3′ (reverse) for *ACTB*. PCR amplification was performed using PrimeSTAR HA Taq polymerase (Takara Bio, Otsu, Japan) in a final volume of 20 µl. The PCR conditions involved initial denaturation at 94°C for 5 min, followed by 40 cycles at 98°C for 10 s, 62°C for 5 s, 72°C for 30 s, and a final extension at 72°C for 10 min. Then, 20 µl of each PCR product was size-fractionated using 2% agarose gel electrophoresis.

### Statistical Analysis

The data obtained in this study were analyzed using the GraphPad Prism statistical program (GraphPad Software, San Diego, CA). Data on developmental rates were arcsine transformed and then examined using analysis of variance (ANOVA) and a Newman–Keuls multiple comparison test. Relative transcription levels between the sexes for each type of embryo or between *in vivo* and in *vitro* embryos were analyzed using unpaired Student's *t* tests; Sc treatment means were compared using ANOVA followed by Dunnett's test. All data are expressed as mean values ± SEM. A probability of *P*<0.05 was considered statistically significant in all tests.
